# A bibliometric analysis of intra-articular injection therapy for knee osteoarthritis from 2012 to 2022

**DOI:** 10.1097/MD.0000000000036105

**Published:** 2023-11-17

**Authors:** Zhiyong Lu, Liangyu Xie, Wenbo Liu, Ziteng Li, Yuanzhen Chen, Gongchang Yu, Bin Shi

**Affiliations:** a Shandong First Medical University (Shandong Academy of Medical Sciences), Shandong Province, China; b Shandong University of Traditional Chinese Medicine, Shandong Province, China.

**Keywords:** bibliometric analysis, CiteSpace, intra articular injection, knee osteoarthritis

## Abstract

Knee osteoarthritis (KOA) is the most common joint disease worldwide and, with the progression of an aging population, is one of the most important causes of disability worldwide. Its main symptoms include articular cartilage damage, periarticular pain, swelling, and stiffness. Intra-articular (IA) injections offer many advantages over systemic administration and surgical treatment, including direct action on the target joint to improve local bioavailability, reduce systemic toxicity, and lower costs. This study analyzed KOA intra-articular injection treatment and its hot literature and research horizons using bibliometric methodologies and graphical tools to aid future research. We performed a bibliometric analysis of 2360 publications in the Web of Science core collection using CiteSpace software. The United States (28.26% of publications) and China (18%) had the biggest publications. Rush University was the most active institution, but Boston University had the greatest citation/publication rate (65.77), suggesting a high literature standard. The majority of publications were in Osteoarthritis and cartilage. Bannuru RR was the most referenced author, while Filardo, Giuseppe was the most productive author. Studies in platelet-rich plasma (PRP), mesenchymal stem cells (MSCs), and microsphere formulation are likely to be future research hotspots. The current scientometric study provides an overview of KOA intra-articular injection therapy studies from 2012 to 2022. This study outlines the current research hotspots and potential future research hotspots in the field of intra-articular injection treatment for KOA and may serve as a resource for researchers interested in this topic.

## 1. Introduction

Knee osteoarthritis (KOA) is the most prevalent joint disease in the world, with an estimated global prevalence of 3.8% and a greater prevalence in women (mean 4.8%) than in males (mean 2.8%) as one of the primary causes of disability globally.^[1]^ KOA is a multifactorial degenerative illness that is commonly characterized by articular cartilage “wear and tear.” Furthermore, it has been demonstrated that KOA is a condition induced by chronic low-grade inflammation.^[2]^ It mainly damages the articular cartilage and causes pain, swelling, and stiffness around the joints.^[3]^ Intra-articular injectable therapy has several advantages over systemic administration and surgical treatment, such as increased local bioavailability, decreased systemic exposure, fewer unpleasant effects, and lower costs.^[4]^ In addition, other structures that make up the joint such as ligaments, synovium, and muscles, play this role in the KOA process,^[5]^ while intra-articular therapy targets the entire joint cavity. Placebo effects can have an impact on the treatment of KOA, and the effect size of intra-articular placebo injections may be greater than that of topical and oral placebo.^[6]^ Therefore, joint cavity injection therapy is a better treatment option than systemic administration.

Intra-articular therapies is a method of drug delivery that has many advantages compared to systemic administration. Firstly, intra-articular delivery can improve local bioavailability. This means that the drug can more directly act on the affected joint, increasing the concentration of the drug in the joint, and thereby more effectively treating joint diseases. Secondly, intra-articular delivery can reduce systemic exposure. As the drug is injected directly into the joint instead of being transported through the systemic circulation system, the concentration of the drug in other parts of the body is reduced, thereby reducing side effects and adverse events on other organs throughout the body. In addition, reducing adverse events is also an advantage of Intra-articular therapies. As the drug acts directly on the joint, it can reduce unnecessary accumulation of the drug in other parts of the body, thereby reducing the occurrence of drug adverse reactions. Finally, intra-articular delivery can also reduce costs. As a smaller dose of the drug is needed compared to systemic administration, the cost of the drug can be reduced. In summary, intra-articular therapies has many advantages, including improving local bioavailability, reducing systemic exposure, reducing adverse events, and reducing costs. Intra-articular therapies include Glucocorticoids, hyaluronic acid (HA), Intra-articular delivery of biologics, Cell therapies, and the intra-articular therapy pipeline.^[7]^ Of these, only glucocorticoids have received the majority of support. Even though HA is widely used in the articular cavity treatment of KOA, there is still controversy about whether to look at HA or not. Other agents also suffer from inconsistent preparation standards or insufficient evidence of their safety and efficacy. Despite the unresolved issues, the prospect of intra-articular injection therapy for KOA continues to interest clinicians and patients.

The bibliometric analysis can summarize the research profile of research teams and individual researchers, thereby revealing research trends in the target field. It also gives a means of visualizing the contributions of academic organizations and individuals.^[8]^ CiteSpace is a widely-used Web-based Java program for data analysis and visualization in bibliometrics.^[9]^ The Leiden University, Netherlands-based VOSviewer version 1.6.18 software was used to build online visualizations of related journals, institutions, and keyword presentations. In this study, we gathered scholarly articles pertaining to KOA intra-articular injection treatment from the core collection of the Web of Science (WOS) database between January 1, 2012, and November 1, 2022. CiteSpace and VOSviewer were utilized to describe the intra-articular injection treatment collar knowledge structure. Our objective is to present a comprehensive review of the current scientific achievements and future developments in intra-articular injection treatment for KOA.

## 2. Material and Methods

### 2.1. Data source

**Figure 1.**
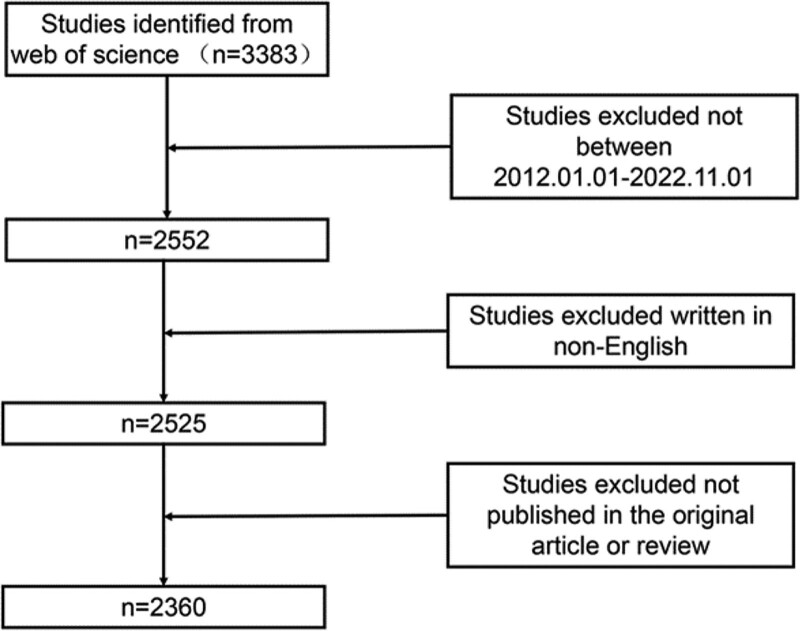
Search strategy.

In total,2552 records were identified after database searching. We conducted the search using Clarivate Analytics’ WOS core database since it is the database of choice for bibliometric analysis.^[10]^ The search technique depicted in Figure 1 was run on November 28, 2022, utilizing the following search formula during an advanced WOS core set search. Ts = (((osteoarthritis, knee OR knee osteoarthritis OR knee osteoarthritis OR KOA OR osteoarthritis of the knee) AND (Intraarticular injection OR Intra-articular Injection))) The inclusion criteria for the literature were as follows: the manuscript was about intra-articular injection treatment for KOA and its complete text was accessible; the document type com-prised articles and reviews; and it was written in English. The following factors determined exclusion: meeting abstracts, editorial materials, correspondences, corrections, and reprints. Finally, 2360 records were obtained.

### 2.2. Research method

The following values were entered into CiteSpace: a time range (2012–2022), a number of years per slice (1), a year of review (LBY = 8), a selection criterion (g-index: k = 25), a minimum duration (MD = 1), and all possibilities from the terminology source, one node type at a time. The findings of our analysis of nation, institution, literature, author, journal, and keyword are presented by the node. The size of the node represents the occurrence frequency; the smaller the node, the lower the occurrence frequency. The connecting lines between the nodes represent collaborative, co-occurrence, or co-citation links. Different colored circles, from inner to outer, represent the years 2012 through 2022. The outermost purple ring signifies that the node has a very high centrality and is typically regarded as an important node in a certain area.^[11]^

## 3. Results

### 3.1. Temporal distribution of literature

From 2012 to November 01, 2022, 2360 publications on KOA joint cavity injection therapy were published on the WOS, including 1905 (80.72%) articles and 455 reviews (19.28%). The literature covers 74 countries or regions and 411 institutions.

**Figure 2.**
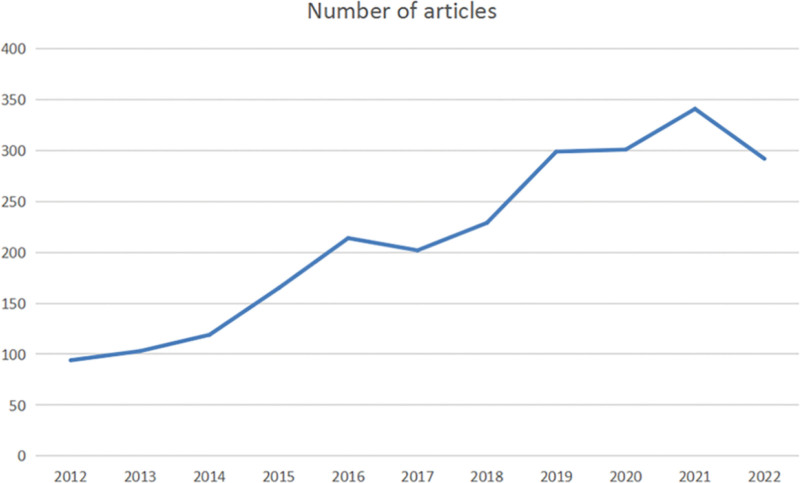
Distribution of publications on knee osteoarthritis (KOA) intra-articular injection therapy studies by year.

Between 2012 and November 1, 2022, 2360 articles related to KOA intra-articular injection treatment were published in the WOS. The annual production from 2012 to 2022 shows a general upward trend in the number of articles related to KOA intra-articular injection treatment (Fig. 2). The number of publications per year was 94 in 2012, 103 in 2013, 119 in 2014, 165 in 2015, 214 in 2016, 202 in 2017, 229 in 2018, 299 in 2019, 301 in 2020, 341 in 2021, and 292 in 2022. The number of papers on intra-articular injection therapy published by KOA Joint in 2021 was the highest in the last decade.

### 3.2. National and institutional literature publication volume

**Figure 3.**
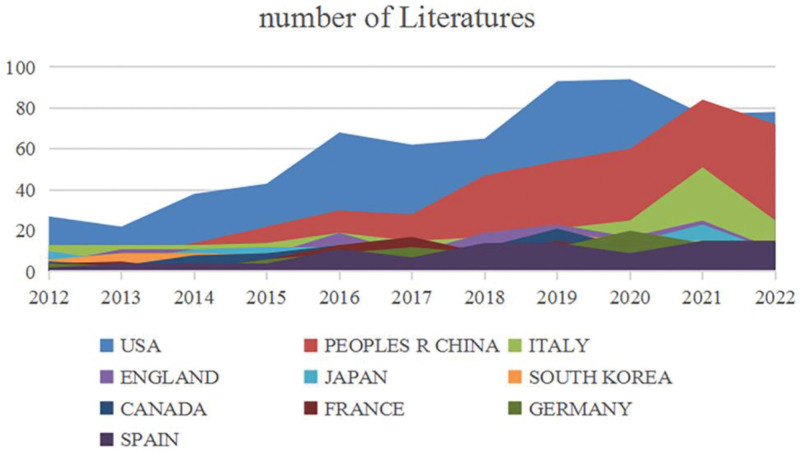
Number of national publications on KOA intra-articular injection therapy studies. KOA = knee osteoarthritis.

**Figure 4.**
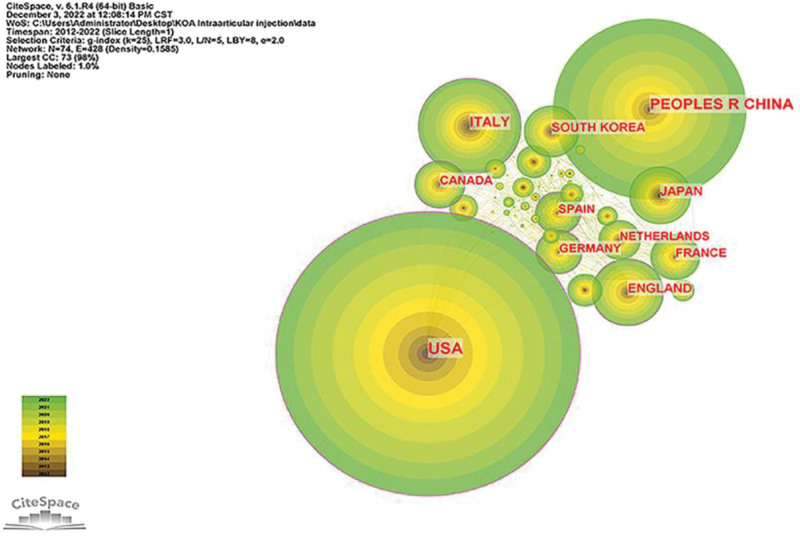
Country co-occurrence map on KOA intra-articular injection therapy studies. KOA = knee osteoarthritis.

Figure 3 depicts the country-by-country publication trends for KOA intra-articular injection treatment from 2012 to 2022. The United States has 667 publications, followed by China with 425, Italy with 226, the United Kingdom with 157, and Japan with 137. Figure 4 shows that the United States has been the most published and referenced country among the top 10 countries/regions over the past decade, showing that it has been a significant force in the growth of KOA intra-articular injection treatment research. Table 1 shows the number of citations per nation. The United States has 20,393 citations, which is much higher than any other nation, and its citation/publication rate is strong (30.57.) Although China has more citations (7768), its citation/publication rate (18.28) is lower than that of other nations. China has a significant performance in terms of publishing production, but its centrality is just 0.07 compared to 0.61 for the United States. The fact that Canada has the greatest citation/publication rate (44.67), although having a relatively modest number of publications, implies that its published articles are of high quality.

**Table 1:** Number of national publications and literature citations on KOA intra-articular injection therapy.

	Country/region	Article count	Percentage (N/2360)	Citations	Citation per publication
1	USA	667	28.26	20393	30.57
2	Peoples R. China	425	18.00	7768	18.28
3	Italy	226	9.58	6190	27.39
4	England	157	6.65	4153	26.45
5	Japan	137	5.80	2954	21.56
6	South Korea	123	5.21	4102	33.35
7	Canada	111	4.70	4958	44.67
8	France	110	4.66	3420	31.09
9	Germany	102	4.32	2285	22.40
10	Spain	99	4.19	3050	30.81

**Figure 5.**
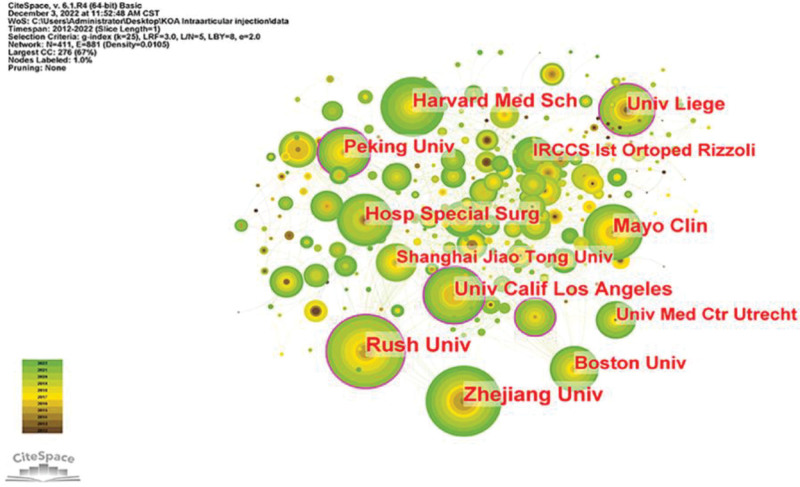
Synoptic diagram of the institution studying intra-articular injection therapy in KOA. KOA = knee osteoarthritis.

**Figure 6.**
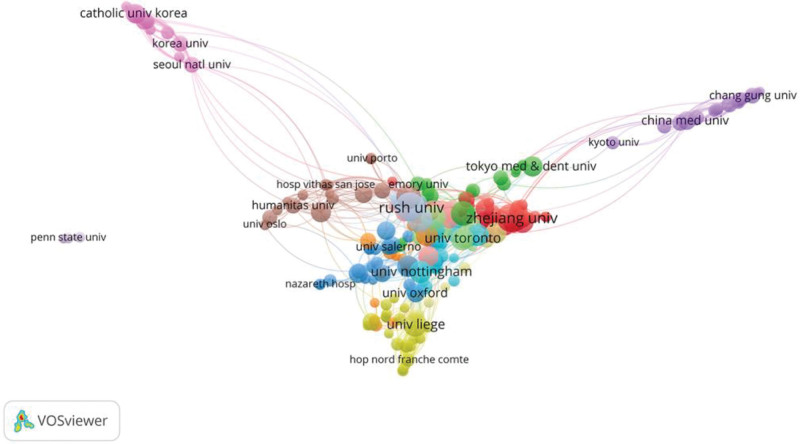
Inter-institutional cooperation diagram for KOA intra-articular injection therapy. KOA = knee osteoarthritis.

The active institutions regarding KOA intra-articular injection therapy research are shown in Figure 5. Table 2 displays the 10 institutions with the most number of publications. Rush Univ published 40 papers with 2395 citations, followed by Zhejiang Univ (39 papers, 1202 citations), Harvard med sch (33 papers, 741 citations), Mayo Clin (31 papers, 1243 citations), and Univ Calif Los Angeles (31 papers, 1243 citations). Consistent with the aforementioned nation study, 6 of the ten most productive institutions were located in China and the United States. Among the top 10 institutions, RUSH Univ had the highest total number of citations (2395) and a high average number of citations (59.88), indicating that RUSH Univ publishes higher-quality articles in this field and can be regarded as a significant center for KOA intra-articular injection therapy research. Figure 6 depicts the institutional cooperation study we did, which reveals the collaboration between institutions. According to the findings, there are essentially no ties between various countries and institutions, and collaboration between institutions is mostly restricted to countries or regions.

**Table 2:** Top 10 high-yield institutions for KOA intra-articular injection therapy studies.

	Institution	Article count	Citations	Citation per publication	Country
1	RUSH Univ	40	2395	59.88	USA
2	ZHEJIANG Univ	39	1202	30.82	China
3	Harvard med sch	33	741	22.45	USA
4	Mayo Clin	31	1243	40.10	USA
5	Univ Calif Los Angeles	31	874	28.19	USA
6	Hosp special surg	29	565	19.48	USA
7	Univ liege	28	832	29.71	Belgium
8	Peking Univ	27	533	19.74	China
9	Boston Univ	26	1710	65.77	USA
10	Shanghai Jiao Tong Univ	22	753	34.22	China

### 3.3. Analysis of journals and co-cited journals

**Figure 7.**
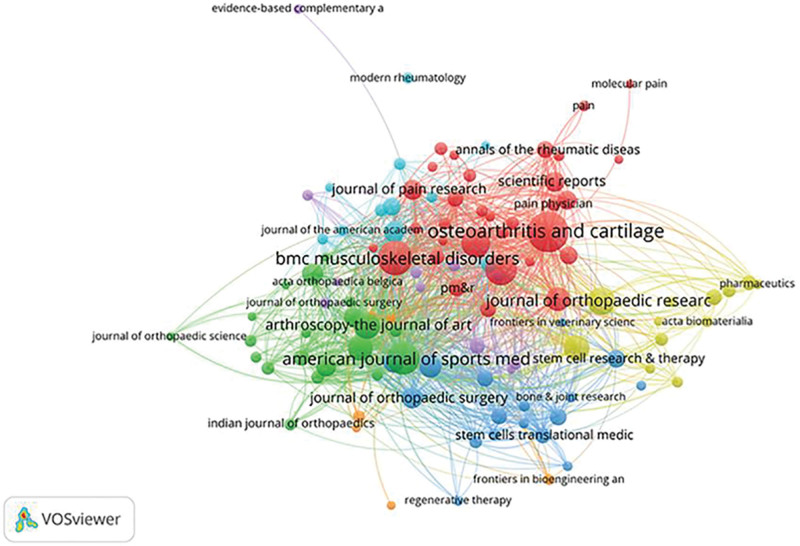
Publication of the journal of KOA intra-articular injection therapy. KOA = knee osteoarthritis.

Figure 7 depicts the periodicals in which the material was published. Table 3 lists the leading 10 publications. Osteoarthritis and cartilage (98 articles, 3.48%) published the most articles in this field among the 10 most prolific journals, followed by Bmc musculoskeletal disorders (72 articles, 3.32%), American Journal of Sports Medicine (65 articles, 3.00%), cartilage (60 articles, 3.00%), and Plos one (50 articles, 2.79%). Osteoarthritis and cartilage was the journal with the highest IF (7.507) and the most citations. The American journal of sports medicine has the greatest rate of citations and publications (54.23). Six of the leading 10 journals were classed as Q1, 3 as Q2, and one as Q3.

**Table 3:** Top ten publications on intra-articular injection therapy for KOA.

	Published journals	Number of articles issued	IF	Quartile in category	Percentage (N/2360)	Total citations	Citation per publication
1	Osteoarthritis and cartilage	98	7.507	Q1	4.15	3746	38.22
2	Bmc musculoskeletal disorders	72	2.562	Q3	3.05	1076	14.94
3	American journal of sports medicine	65	7.010	Q1	2.75	3525	54.23
4	cartilage	60	3.117	Q2	2.54	700	11.67
5	Plos one	50	3.752	Q2	2.11	965	19.3
6	Journal of orthopedic research	45	3.102	Q2	1.90	991	22.02
7	Arthroscopy-the journal of arthroscopic and related surgery	41	5.973	Q1	1.74	2171	52.95
8	International journal of molecular sciences	39	6.208	Q1	1.65	517	13.26
9	Knee surgery sports traumatology arthroscopy	38	4.114	Q1	1.61	1481	38.97
10	Arthritis research & therapy	35	5.606	Q1	1.48	1259	35.97

Table 4 lists the ten most-cited journals discussing intra-articular injection treatment for KOA. The journal with the most co-citations was Osteoarthr cartilage (1986), followed by Ann rheum dis (1371 citations). Ann rheum dis was referenced 3640 times and had the greatest impact factor (IF) (27,973) among the top 10 co-cited journals.

**Table 4:** Top 10 co-cited journals on KOA intra-articular injection therapy.

	Co-cited journals	Citations	Quartile in category
1	Osteoarthr Cartilage	1986	Q1
2	Ann Rheum Dis	1371	Q1
3	Arthritis Rheum-us	1192	Q1
4	J Bone Joint Surg Am	1080	Q1
5	Arthritis Res Ther	1051	Q1
6	J Rheumatol	975	Q2
7	Am J Sport Med	954	Q1
8	Arthritis Rheum	923	Q1
9	Bmc Musculoskel Dis	882	Q3
10	Arthroscopy	821	Q1

### 3.4. Author and co-citation author analysis

**Figure 8.**
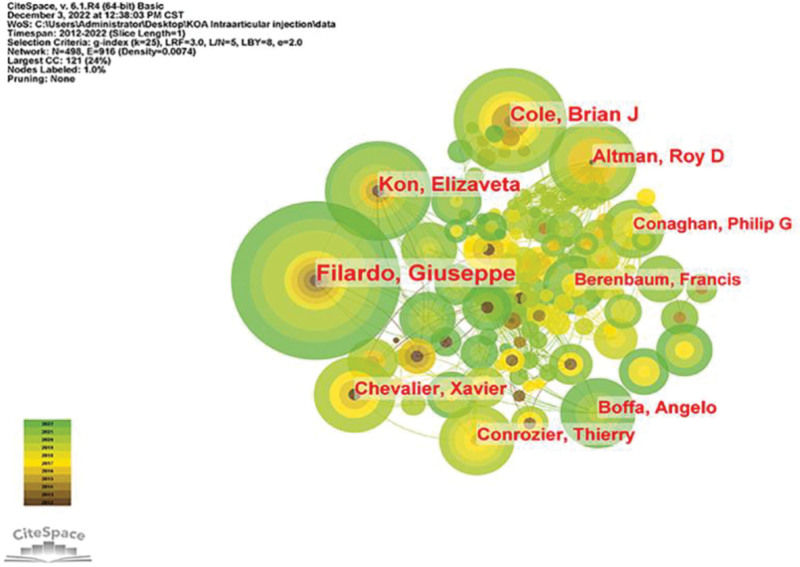
Posting author.

**Figure 9.**
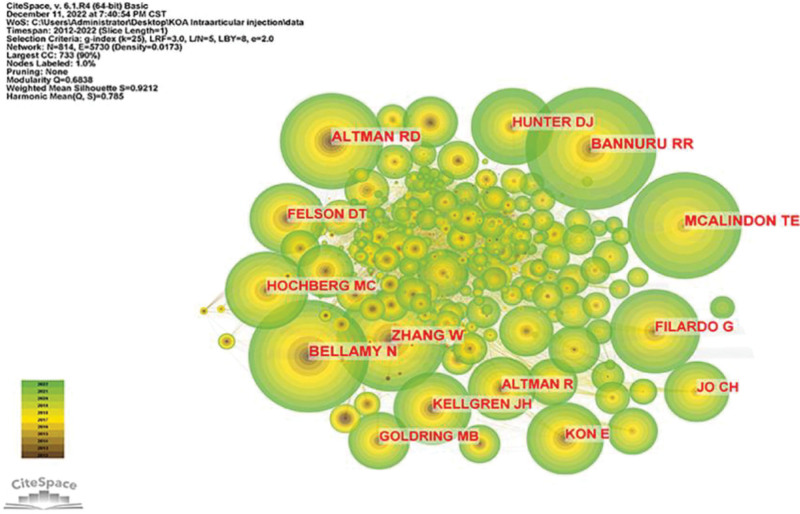
Co-cited authors.

All authors who published literature related to KOA intra-articular injection therapy in the last decade are shown in Figure 8, and the 10 most productive authors are shown in Table 5. By analysis, Filardo, and Giuseppe published the most papers with a total of 27 articles, followed by Cole, Brian J (18 publications) and Kon, Elizaveta (17 publications). Further analysis shows that 4 of the top ten authors are from Italy, 3 from France, 2 from the USA, and one from the UK. Co-cited authors are shown in Figure 9, where the number of authors’ citations correlates with the size of the nodes, including Bannuru RR (455Citation), Bellamy N (419Citation), Mcalindon TE (394Citation), Zhang W (385Citation) and Altman RD (360Citation)

**Table 5:** Authors and co-cited authors.

	Author	Number	Co-cited authors	Number of citations
1	Filardo Giuseppe	27	Bannuru R.R.	455
2	Cole Brian J.	18	Bellamy N.	419
3	Kon Elizaveta	17	Mcalindon T.E.	394
4	Altman Roy D.	14	Zhang Wei	385
5	Chevalier Xavier	13	Altman R.D.	360
6	Boffa Angelo	12	Filardo G.	316
7	Conrozier Thierry	12	Hochberg M.C.	291
8	Maffulli Nicola	10	Hunter D.J.	290
9	Conaghan Philip G.	10	Kellgren J.H.	275
10	Berenbaum Francis	10	Kon E.	274

### 3.5. Analysis of cited references

**Figure 10.**
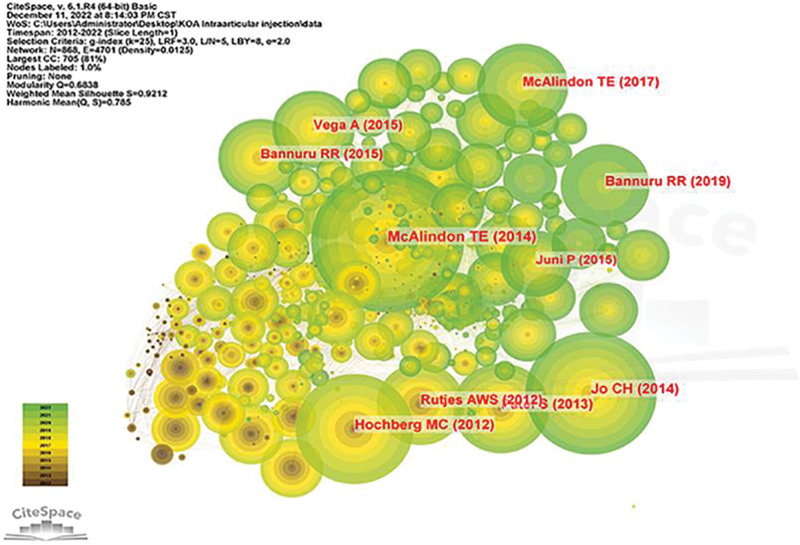
Co-cited references.

With a 1-year time slice and a 2012 to 2020 coverage span, the co-citation network graph had 868 nodes and 1524 linkages (Fig. 10). According to the 5 articles most commonly mentioned (Table 6), the OARSI guidelines for the non-surgical management of KOA (with 241 co-citations) published in Osteoarthritis Cartilage (IF = 7.507) was the most cited article by Mcalindon TE. Also, among the top 5 co-cited papers, Mcalindon TE authored Effect of Intra-articular Triamcinolone vs Saline on Knee Cartilage Volume and Pain in Patients With KOA: A Randomized Clinical Trial, which was published in JAMA.

**Table 6:** Top 5 co-cited references.

	Title	Journals and IF	DOI	Publication time	Total number of citations
1	OARSI guidelines for the non-surgical management of knee osteoarthritis	Osteoarthritis Cartilage (IF = 7.507)	10.1016/j.joca.2014.01.003	2014.04	241
2	Intra-articular injection of mesenchymal stem cells for the treatment of osteoarthritis of the knee: a proof-of-concept clinical trial	Stem Cells (IF = 5.845)	10.1002/stem.1634	2014.05	207
3	American College of Rheumatology 2012 recommendations for the use of nonpharmacologic and pharmacologic therapies in osteoarthritis of the hand, hip, and knee	Arthritis Care Res (Hoboken) (IF = 5.178)	10.1002/acr.21596	2012.04	186
4	Treatment with platelet-rich plasma is more effective than placebo for knee osteoarthritis: a prospective, double-blind, randomized trial	Am J Sports Med (IF = 7.010)	10.1177/0363546512471299	2012.02	152
5	Effect of intra-articular triamcinolone vs saline on knee cartilage volume and pain in patients with knee osteoarthritis: a randomized clinical trial	JAMA (IF = 157.335)	10.1001/jama.2017.5283	2017.05	143

**Figure 11.**
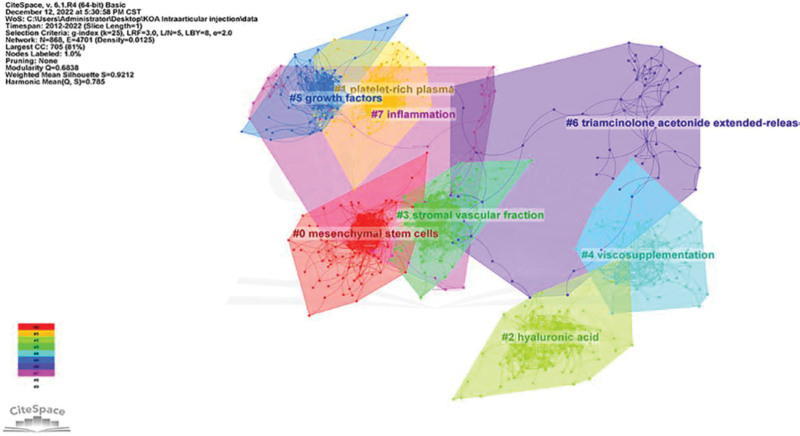
Clustering of co-cited references.

Reference Co-citation Analysis explores the research hotspots in a given domain. Previous studies have shown that LLR labels have high confidence in covering topics with a profile value (0.9212) > 0.7, indicating clustering. Co-cited references were plotted, and 8 major clusters were selected for articles related to KOA intra-articular injection treatment (Fig. 11), including “mesenchymal stem cells,” “platelet-rich plasma,” “hyaluronic acid,” “stromal vascular fraction,” “viscosupplementation,” “growth factors,” “triamcinolone acetonide extended-release,” “inflammation.”

We discovered that 7 of the top ten most-cited papers were associated with “viscosupplementation,” “hyaluronic acid,” and “triamcinolone acetonide extend-ed-release,” indicating the significance of hyaluronic acid (viscosupplementation) and “triamcinolone acetonide extended-release” in this field. This indicates the importance of “triamcinolone acetonide extended-release” in this field. One paper investigated the therapeutic effect of PRP in early KOA, and 2 papers evaluated the therapeutic effect of intra-articular injection of autologous MSCs in KOA, indicating that MSC therapy has received widespread attention for its safety and efficacy as a novel therapeutic modality in the treatment of KOA by intra-articular injection. By analyzing the co-cited literature, we conclude that glucocorticoids and HA are the most widely used treatment modalities in the intra-articular treatment of KOA.

**Figure 12.**
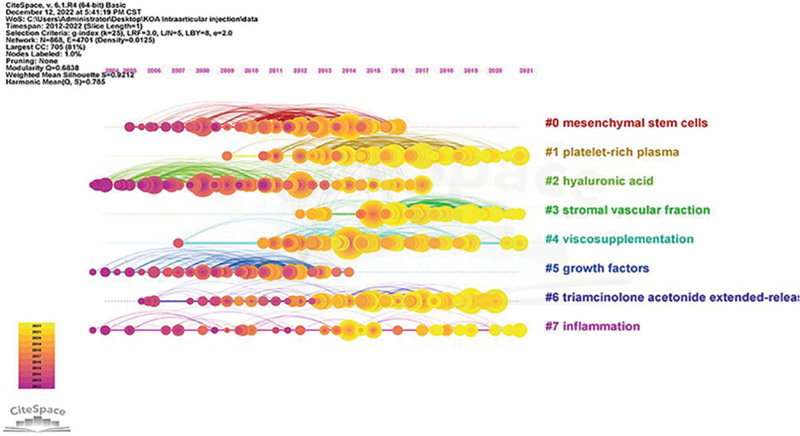
Timeline of co-cited references.

A timeline view was drawn for all clusters using CiteSpace. The publication date is at the top, and the most recent publication is on the right (Fig. 12). The results show that “hyaluronic acid,” “growth factors,” and “inflammation” appear first, and the current study focuses more on “platelet-rich plasma,” “stromal vascular fraction,” and “triamcinolone acetonide.” The current study focuses more on “platelet-rich plasma,” “stromal vascular fraction,” and “triamcinolone acetonide extended-release,” which may be the focus of future studies.

**Figure 13.**
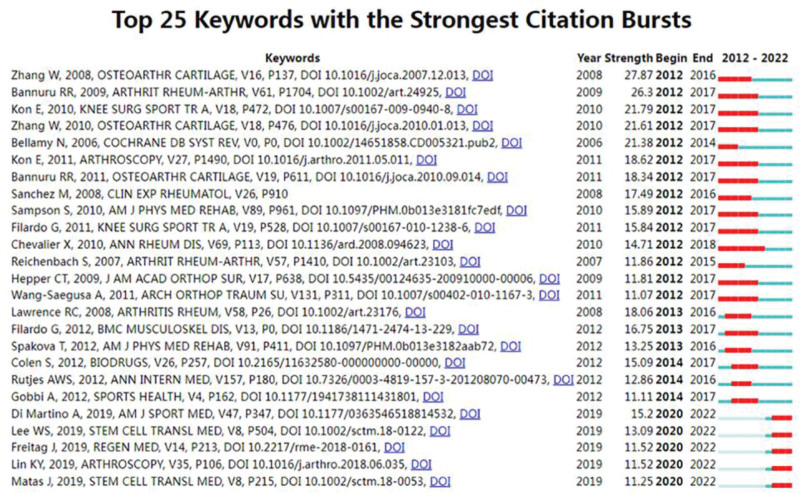
Co-cited references burst.

We used CiteSpace to map the top 25 citations for KOA articular cavity injection therapy from 2012 to 2022 based on a burst detection algorithm (Fig. 13). OARSI recommendations for the management of hip and KOA, Part II: OARSI evidence-based, expert consensus guidelines the highest intensity was 27.87. Secondly, Therapeutic trajectory of hyaluronic acid versus corticosteroids in the treatment of KOA: a systematic review and meta-analysis (Intensity 26). Platelet-rich plasma: intra-articular knee injections produced favorable results on degenerative cartilage lesions (Intensity 21.79). While Single, intra-articular treatment with 6 ml Hylan G-F 20 in patients with symptomatic primary osteoarthritis of the knee: a randomized, multicenter, double-blind, placebo-controlled trial outbreak duration up to 6 years (2012–2018), these high-intensity outbreaks in the literature reaffirm the extensive research on hyaluronic acid versus corticosteroids in the treatment of osteoarthritis of the knee, in line with our previous findings.

### 3.6. Keyword co-occurrence analysis

**Figure 14.**
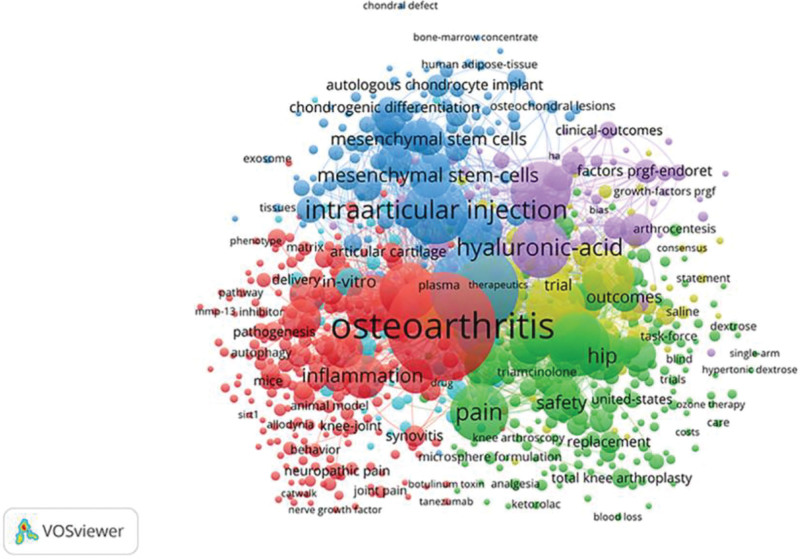
Keyword co-occurrence network.

The examination of keyword co-occurrence is essential for the investigation of article subjects. Using VOSviewer, we created a visual network diagram of the keywords (Fig. 14). 756 of the 5897 keywords happened 5 times or more. There was a significant correlation between the terms “osteoarthritis” (1289 occurrences, 11,465 total link strength), “knee osteoarthritis” (856 occurrences, 7733 total link strength), “knee” (544 occurrences, 5046 total link strength), “intraarticular injection” (460 occurrences, 4462 total link strength), and “efficacy” (424 occurrences, 4175 total link strength).

**Figure 15.**
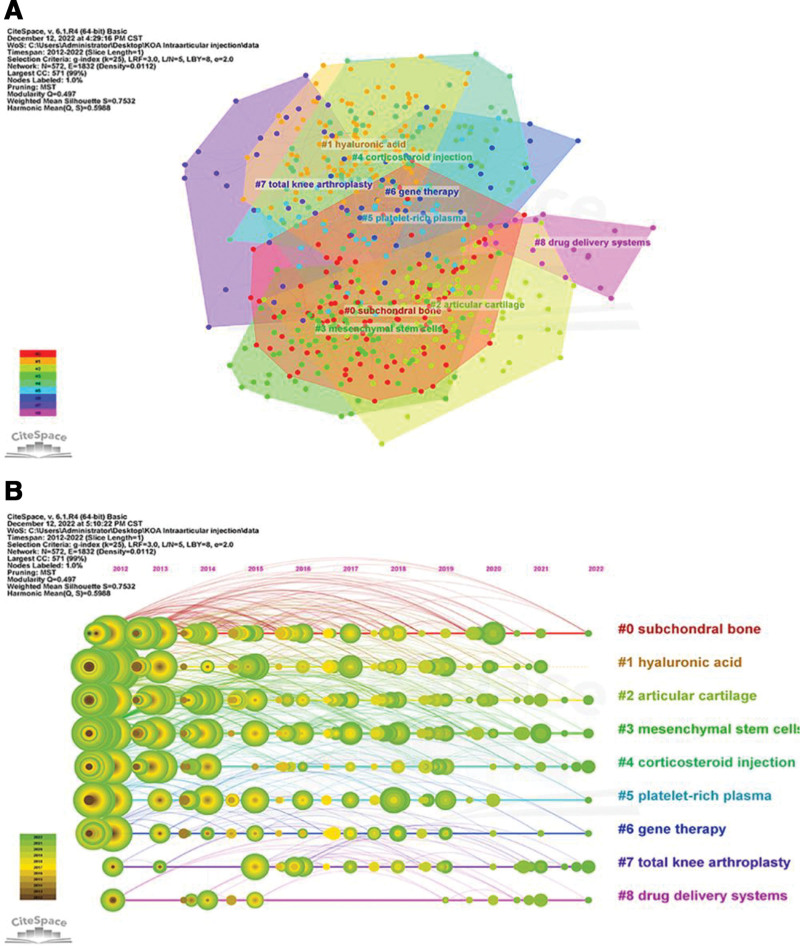
(A) Clustering analysis of correlates. (B) Keyword clustering timeline diagram.

We constructed a keyword clustering viewable through CiteSpace (Fig. 15A) and plotted a timeline view of keyword clustering (Fig. 15B). We found that “subchondral bone,” “hyaluronic acid,” “articular cartilage,” “mesenchymal stem cells,” “corticosteroid injection,” and “platelet-rich plasma” are gradually increasing the number of studies related to these fields. In recent years, the field represented by these clusters has become a prominent study focus, as shown by the bigger nodes and warmer hues. This is consistent with the emphasis on highly referenced material discussed above.

To investigate the research hotspots in the field of KOA intra-articular injection therapy, keywords were extracted from the 2360 publications for co-occurrence analysis on CiteSpace software. Table 7 shows the top 10 highest-frequency and highest-centrality keywords. Among these keywords, “knee osteoarthriti” (857 times) frequently appeared, followed by “intraarticular injection” (725 times), “hyaluronic acid”(686 times), “platelet-rich plasma (prp)” (428 times), “efficacy” (413 times), and “articular cartilage” (389 times). In terms of centrality, “knee osteoarthriti” (0.41) was the most central, followed by “intraarticular injection” (0.26), “hyaluronic acid” (0.14), “therapy” (0.13), and “double blind” (0.09).

**Table 7:** The top 10 keywords for the highest count and centrality on KOA intra-articular injection therapy.

	Count	Keywords	Centrality	Keywords
1	857	Knee osteoarthriti	0.41	Knee osteoarthriti
2	725	Intraarticular injection	0.26	Intraarticular injection
3	686	Hyaluronic acid	0.14	Hyaluronic acid
4	428	Platelet-rich plasma (prp)	0.13	Therapy
5	413	Efficacy	0.09	Double blind
6	389	Articular cartilage	0.08	Articular cartilage
7	380	Double blind	0.07	Efficacy
8	369	Osteoarthriti	0.06	Knee
9	288	Knee	0.05	Platelet-rich plasma (prp)
10	274	Management	0.04	Osteoarthriti

**Figure 16.**
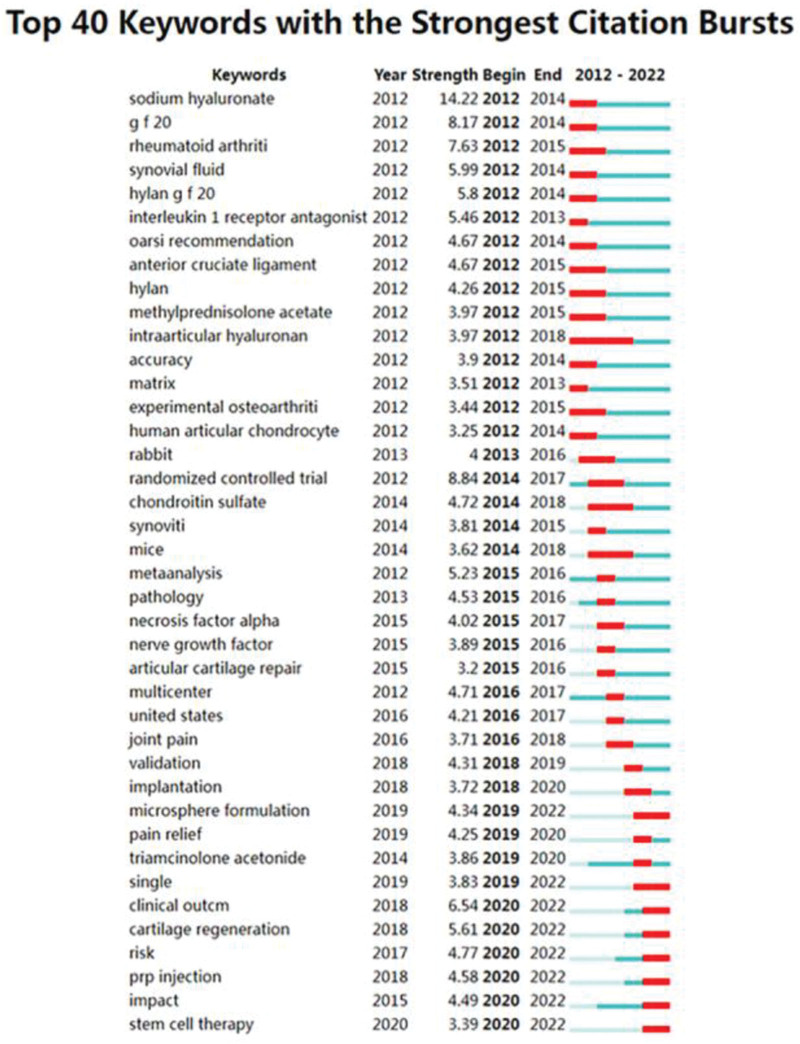
Keyword burst.

The keyword burst detection algorithm identifies the most popular keywords in recent years. The keyword “sodium hyaluronate” has the largest occurrence value of 14.22. “Intraarticular hyaluronan” has the longest occurrence time, from 2012 to 2018. We focused on keywords that have been in the outbreak since (Fig. 16), including “microsphere formulation” (outbreak intensity of 4.34), “single” (outbreak intensity of 3.83), “clinical outcome” (outbreak intensity of 6.54), “cartilage regeneration” (outbreak intensity of 5.61), “risk ‘ (burst intensity of 4.77) ‘PRP injection’ (burst intensity of 4.58) ‘impact’ (burst intensity of 4.49)’ stem cell therapy” (burst intensity of 3.39). It may be a hot spot for future research.

## 4. Discussion

### 4.1. Research hotspots

Hot topics in this field are examined via keyword clustering analysis. Herein, keyword clustering analysis revealed main hot topics in KOA intra-articular injection therapy research: “subchondral bone,” “hyaluronic acid,” “articular cartilage,” “mesenchymal stem cells,” “corticosteroid injection,” and “platelet-rich plasma.”

Under normal culture circumstances, “mesenchymal stem cells” (MSCs) are defined as adhering cells with a spindle-shaped morphology specified by the International Society for Cellular Therapy in 2006. They express CD105, CD73, and CD90, but not CD45, CD34, CD14, CD11b, CD79a, CD19, or HLA-DR, and may develop in vitro into osteoblasts, adipocytes, and chondrogenic cells. These requirements, however, are insufficient to characterize MSCs.^[12]^ Although the initial notion of MSCs pertains exclusively to cells in the BM (bone marrow stromal cells), its therapeutic use as an intra-articular treatment for OA can be expanded to include cells from various sources (e.g., synovium, adipose tissue, dental pulp, etc).^[7]^ Therapy using mesenchymal stem cells (MSCs) is a treatment for KOA with high hopes. Studies have established that the in vitro development of MSCs has tremendous potential for treating a variety of tissue damage, organ failure, inflammatory illnesses, and autoimmune disorders^.[13]^ It was thought that stem cells could stimulate cartilage repair, delaying or preventing the need for total knee arthroplasty.^[14]^ Regarding the efficacy of MSCs in the treatment of KOA, a study revealed that cell doses of 10, 40, and 100 million autologous cells per knee joint resulted in very similar healing outcomes and that the majority of the effects obtained 1 year after cell application persisted 2 and 4 years later.^[15]^ Individuals with KOA level 2 were most likely to exhibit the optimal response with 40 106 cells. Similarly, considerable improvements were observed with both low (24 106) and high (100 106) cell counts, but there may be no response with chronic discomfort or edema.^[16]^ Numerous European research and clinical trials indicate that bone marrow MSCs are the primary source of cells; nonetheless, the ideal source of MSCs remains speculative when taking into account the cell preparation procedure, differentiation capacity, and enduring effects of MSCs. Successful use of in vitro expanded MSCs may need optimization of aspects relating to MSC production, manipulation, assessment of the immunological state of the recipient, and delivery route and dosage. Importantly, additional concurrent medications should not interfere with the desirable immuno-modulatory effect of MSCs.^[17]^

Platelet-rich plasma (PRP) is a patient-derived blood product. The physician takes a minute quantity of peripherally sedated blood, processes it by centrifugal concentration, and reinjects it into the patient joint cavity.^[18]^ Platelets contain 3 distinct types of granules:α -granules, dense granules, and lysosomal granules. Vascular endothelial growth factor, platelet-derived growth factor, insulin-like growth factor-1, and transforming growth factor are all produced by -granules.^[19]^ Additionally, other factors in alpha particles, such as coagulation factors and chemokines, have been shown to stimulate chondrocyte and cartilage MSC proliferation, promote chondrocyte cartilage matrix secretion, and attenuate the catabolic effects of proinflammatory cytokines, thus positively influencing the osteoarthritic state.^[20,21]^

At 12 months, there were no significant differences in joint structure or symptoms between intra-articular PRP injections and saline placebo injections in patients with symptomatic mild-to-moderate MRI knee OA. These findings do not support using PRP to treat KOA.^[22]^ In a controlled study with HA, both groups were observed for an average of 11.1 months. The mean improvement in total WOMAC score was much greater in the PRP group (44.7% vs 12.6%) than in the HA group (P.01). Patients with knee OA who are treated with PRP may see better outcomes than those who get HA. Leukocyte-poor PRP (LP-PRP) may be a more effective treatment option than leukocyte-rich PRP (LR-PRP) for KOA.^[23]^ One of the most disputed issues concerning the efficacy of PRP is whether the presence of leukocytes is detrimental.^[24]^ Leukocytes in PRP, according to Wen-Jing Yin, produce an increase in proinflammatory cytokines and enzymes that may have an opposite effect.^[25]^ To address this issue, Alessandro Di Martino observed that 3 intra-articular LR-PRP or LP-PRP injections produced equal clinical improvement during a 12-month follow-up period in patients with symptomatic knee OA. In both treatment groups, the frequency of adverse events was low, and there were no significant differences between groups. It was established that the presence of leukocytes did not affect the clinical outcome of PRP injections considerably.^[26]^

In clinical research, methodologies for preparing PRP have been described inconsistently, and the majority of studies have not supplied sufficient information to duplicate the protocol. In addition, available data on the manufacturing and content of PRP do not allow for a comparison of PRP products provided to patients. There is a need for comprehensive, precise, and exhaustive descriptions of PRP preparation processes^[27]^ in order to simplify comparisons between studies and assure their reproducibility.

It is obvious that glucocorticoids and HA are now the most widely used intra-articular (IA) treatments, despite the fact that perspectives about mucus supplementation vary by country.^[28]^ The AAOS intended to conduct a network meta-analysis to compare the clinical efficacy of NSAIDs, acetaminophen, intra-articular corticosteroids, intra-articular PRP, and intra-articular hyaluronic acid versus placebo. Intra-articular HA did not score among the top 5 therapies in terms of pain, function, and the combination of pain and function. In the pain trial, intra-articular HA scored the highest, ranking sixth. Given the lack of therapeutic effect, intraarticular hyaluronic acid is not recommended for symptomatic knee OA^.[29]^

According to a study,^[30]^ HA may have antioxidant and anti-apoptotic effects and reduce inflammation by modulating the AKT pathway. In addition, HA has lubricating, analgesic, and possibly tissue-repair characteristics.^[31]^ It has been discovered that IA hyaluronic acid is employed often in mild to severe OA.^[32]^ Therapeutic trajectory following intra-articular hyaluronic acid injection in KOA - meta-analysis yielded the same conclusion, namely that IAHA is efficacious at 4 weeks, reaches maximum efficacy at 8 weeks, and exerts detectable residual effects at 24 weeks.^[33]^ In addition, a number of studies^[34]^ have investigated whether HA can attenuate the cytotoxic effects of glucocorticoids and anesthetics. One study examined the efficacy and safety of Diclofenac-Hyaluronate (DF-HA) Conjugate (Diclofenac Etalhyalu-rate) in the treatment of KOA utilizing DF covalently linked to a high molecular weight fermented HA. Results demonstrate that when DF-HA is paired with the advantages of IA HA and NSAIDs, symptoms are significantly reduced. To maintain efficacy, DF-HA requires repeated injections every 4 weeks, which may be more expensive than traditional IA formulations, which are administered every 3 to 6 months.^[35]^ Despite its faults, this may indicate a new approach for HA.

KOA is characterized by articular cartilage degeneration and subchondral bone changes.^[36]^ Therefore, while examining the beginning and course of KOA, the origin of the subchondral bone cannot be neglected. Subchondral lesions are linked to symptomatic knee OA.^[37]^ Under the influence of biochemical and mechanical factors, subchondral bone and cartilage from a bone-chondral monolithic functional complex^[38]^ are involved in the etiology of osteoarthritis. In early OA, the self-repair of articular cartilage reduces the excessive mechanical strain on the subchondral bone. As a result, the subchondral bone is not subjected to the necessary stress. In turn, this under load increases the ratio of receptor activator of nuclear factor-kappa B ligand/osteoprotegerin receptor activators in osteocytes, resulting in an increase in osteoclast formation and bone resorption activity^.[39]^ On the one hand, as KOA progresses, the cartilage layer seems abnormally altered, leading to a diminished capacity to absorb mechanical stress and an increase in subchondral bone loading. The rapid turnover of subchondral bone, on the other hand, results in altered biomechanical properties of bone tissue in early OA, transmitting shear loads to the cartilage layer and causing persistent cartilage damage.^[37]^ The subchondral bone plate and subchondral trabecular bone make up the subchondral bone (SB). As osteoarthritis (OA) progresses, uncoupled bone remodeling occurs in the SB, leading to the production of microfractures and vascular infiltration in the subchondral bone plate.^[40]^ Media generated by osteoclasts and chondrocytes can scatter and travel through micro-fissures or invasive arteries.

“Triamcinolone acetonide extended-release.” Corticosteroid intra-articular injection is the most well-approved therapy technique for KOA joint cavity injection. The OARSI recommendations for non-surgical KOA treatment propose intra-articular (IA) corticosteroid injections.^[41]^ According to studies, synovial inflammation plays a significant part in the pathogenesis of KOA. Therefore, the synovium may be an important target in the therapy of KOA,^[42]^ and intra-articular injections of corticosteroids (such as tretinoin) may alter synovial inflammation.^[43]^ Injections of intra-articular tretinoin can alleviate moderate to severe pain,^[44]^ however, the effect declines soon following the injection.^[45]^ A recently developed micro-sphere-based prolonged-release tretinoin formulation is able to sustain tretinoin concentrations in the joint for a longer period of time. Philip G. Conaghan examined the therapeutic effectiveness of FX006 for the treatment of KOA pain and found that FX006 32 mg considerably outperformed the saline group.^[46]^ Uncertain is the benefit of FX006 over traditional tretinoin preparations.

“Inflammation.” Osteoarthritis is an inflammatory disorder. Studies have shown that synovitis is widespread in osteoarthritic joints and that inflammation is present in the synovium at all phases of OA, including the earliest stages.^[47]^ Clinical and epidemiological studies^[48]^ indicate a correlation between the progression of cartilage loss and inflammation in the knee joint of people with KOA. Surface pattern recognition receptors (PRRs) on macrophages, which are germline-encoded innate immune receptors, are activated during the development of osteoarthritis (OA), allowing synovial macrophages to differentiate between external pathogen-associated molecular patterns and endogenous damage-associated molecular patterns. Activated PRRs induce intracellular downstream effects, such as the NF-B pathway, resulting in the production of proinflammatory cytokines and chemokines.^[49]^ il-1 is a proinflammatory cytokine that plays an important role in OA pathogenesis by stimulating OA pathogenesis-associated inflammatory factors such as inducible nitric oxide synthase and cyclooxygenase-2, thereby reducing aggregated glycans and collagen II and promoting ECM degradation.^[50]^ Under inflammatory conditions, chondrocytes undergo metabolic changes mediated by NF-B activation, and according to studies, chronic NF-B activation is one of the key causes of pathological abnormalities in OA. In chondrocytes, mechanical stress, injury, and age, as well as metabolic disease factors, can activate NF-B to promote catabolic alterations. In turn, cartilage degeneration exacerbates mechanical stress and joint injury, exacerbating inflammatory stimulation of the synovial cavity, sustaining NF-B activity, and generating a positive feedback loop.^[51]^

### 4.2. Research frontiers

“Keyword burst” refer to words that are frequently cited over a period of time. The distribution of keywords with the strongest citation burst can be used to predict the frontier of research trends. Through analysis, we believe that Regarding knee joint injection therapy, cartilage repair may receive more attention. PRP, MSCs, and non-steroidal drugs are used as injection materials, but the effective ingredients may rapidly decline over time after injection. Microsphere may slow down this process. Researchers hope to combine PRP, MSCs, and microsphere to repair articular cartilage and alleviate pain caused by KOA.

Alessandro Di Martino et al undertook a 5-year, double-blind, randomized, controlled experiment comparing PRP injections to HA injections for KOA. The median duration of subjective symptom alleviation was 9 months for HA and twelve months for PRP (insignificant). The only meaningful difference was the much-decreased reintervention rate at 24 months in the PRP group. Both therapies enhanced the knee functionality and alleviated its discomfort over time. PRP did not produce greater clinical improvement in symptom function at various follow-up points or duration of impact when compared to HA.^[52]^ In the treatment of mild to moderate KOA, intra-articular injections of PRP were better than HA or saline solution. Individuals with mild to severe KOA who receive intra-articular injections of LP-PRP demonstrate clinically significant functional improvement for a minimum of 1 year.^[53]^ Mengjiao Ma et al loaded PRP powder into polyethylene glycol microspheres, and platelet aggregation prolonged the duration of PRP release from microspheres. This delivery system can be used to load and release PRP to promote tissue regeneration and wound healing, or inhibit tissue degradation of osteoarthritis and disc degeneration.^[54]^

Based on chitosan (CS) hydrogel/3d printed polycaprolactone (PCL) hybrid material, pinxue Li et al injected tetrahedral framework nucleic acid into the articular cavity. As a promising DNA nanomaterial to improve the regenerative microenvironment, tetrahedral framework nucleic acid can be absorbed into bone marrow mesenchymal stem cells and promote the proliferation and chondrogenic differentiation of bone marrow mesenchymal stem cells.^[55]^ Six months after a single injection of AD-MSCs, WOMAC scores had significantly improved. Six months later, the WOMAC score of the control group did not change appreciably. At 6 months, MRI demonstrated no change in cartilage abnormalities in the MSC group, but cartilage defects increased in the control group. This suggests that MSCs may influence cartilage healing. At the 6-month follow-up, neither group had unfavorable results. For studies to provide greater evidence, larger sample sizes and longer follow-up periods may be necessary.^[56]^ Julien Freitag applied a semi-quantitative and verified MOAKS evaluation to define structural changes over 12 months and demonstrated a trend toward enhanced disease stability with the twofold injection regimen in comparison to the single injection regimen. The discovery of cartilage stability as opposed to regeneration suggests that MSCs function via paracrine and local support pathways as opposed to direct differentiation along the chondrocyte lineage.^[57]^

In conclusion, PRP and MSCs may be at the forefront of joint injection research for KOA. We highlighted the phrases “microsphere formulation,” “single,” “clinical outcome,” “cartilage regeneration,” “risk,” “PRP injection,” “effect,” and “stem cell treatment” since the outbreak has persisted to date. These keywords are similar to those found in the previously quoted article outbreak and concentrate on the clinical efficacy and safety of PRP and MSCs. The concept of new drug delivery system has shown practical benefits as a new way of accurate, safe and high-quality drug delivery for OA treatment. With the progress of material technology, “microsphere formulation” may become a new trend in the development of corticosteroid drugs for combined residence time and reducing systemic exposure, thereby improving the therapeutic effect of KOA. In addition, herbal medicine is also used to treat osteoarthritis because of its less efficacy and side effects. Herbal medicine combined with microsphere preparation may have better effect.

## 5. Conclusion

The number of papers on the topic of KOA intra-articular injection treatment is increasing year by year. Research disciplines are accompanied by crossover and convergence. The United States is the largest contributor to research on this topic, with the highest number and centrality of studies in the world. There is a need for more inter-country collaboration among authors and institutions of published studies. Mesenchymal stem cells, PRP, hyaluronic acid, triamcinolone acetonide extended-release, inflammation, and subchondral bone are the main research hotspots. From the above analysis, we believe that PRP, MSCs, and microsphere formulation have a broader future for KOA intra-articular injection treatment, but as an emerging therapeutic modality, questions about its efficacy and risk profile still need to be addressed, requiring researchers to conduct more precise experiments and to enhance cooperation among various organizations, institutions, and countries. This requires researchers to conduct more precise experiments and increase cooperation among organizations, institutions, and countries to reach a consensus on the preparation, efficacy, and safety of joint cavity injection reagents.

PRP therapy has shown promise for treating joint problems. PRP is a concentration of blood platelets containing growth factors that can help stimulate the body natural healing processes. Some studies have shown that PRP can promote the repair of cartilage and help relieve pain caused by KOA.MSCSs (mesenchymal stem cells) are a type of adult stem cell that can differentiate into various types of cells, including cartilage cells. Researchers are exploring the use of MSCSs to repair joint cartilage and relieve pain caused by KOA. Microspheres are small, sphere-shaped particles that can carry drugs or growth factors to the joint. By combining PRP, MSCSs, and microsphere formulations, researchers hope to develop a more effective treatment for joint cartilage repair and pain relief in patients with OA. However, it is important to note that research in this area is ongoing, and the long-term effectiveness and safety of these treatments need to be further evaluated. Additionally, the use of these therapies may be limited by factors such as cost and availability.

## 6. Advantages and limitations

This research represents groundbreaking investigation into visual analysis within the field of KOA intra-articular injection treatment, employing a bibliometric approach to comprehend trends and focal points. Despite the implementation of a comprehensive and meticulously organized research methodology, solely articles that were published within the Web of Science Core Collection were chosen for data analysis. This approach may result in the exclusion of pertinent papers. The study only considered English articles, potentially introducing source bias. Subsequent analyses will incorporate additional databases to overcome this constraint.

## Acknowledgments

The authors would like to express their appreciation to Professor C.M. Chen, who invented CiteSpace, which is free to use.

## Author contributions

**Conceptualization:** Zhiyong Lu, Liangyu Xie, Wenbo Liu, Ziteng Li.

**Data curation:** Zhiyong Lu.

**Formal analysis:** Zhiyong Lu, Liangyu Xie.

**Visualization:** Zhiyong Lu.

**Writing – original draft:** Zhiyong Lu.

**Writing – review & editing:** Yuanzhen Chen, Gongchang Yu, Bin Shi.

## References

[1] Cross M Smith E Hoy D. The global burden of hip and knee osteoarthritis: estimates from the global burden of disease 2010 study. Ann Rheum Dis. 2014;73:1323–30. 24553908 10.1136/annrheumdis-2013-204763

[2] Koh SM Chan CK Teo SH. Elevated plasma and synovial fluid interleukin-8 and interleukin-18 may be associated with the pathogenesis of knee osteoarthritis. Knee 2020;27:26–35. 31917106 10.1016/j.knee.2019.10.028

[3] Jang S Lee K Ju JH. Recent updates of diagnosis, pathophysiology, and treatment on osteoarthritis of the knee. Int J Mol Sci . 2021;22:2619. 33807695 10.3390/ijms22052619 PMC7961389

[4] Emami A Tepper J Short B. Toxicology evaluation of drugs administered via uncommon routes: Intranasal, intraocular, Intrathecal/Intraspinal, and Intra-Articular. Int J Toxicol. 2018;37:4–27. 29264927 10.1177/1091581817741840 PMC5874330

[5] Lories RJ Luyten FP. The bone-cartilage unit in osteoarthritis. Nat Rev Rheumatol. 2011;7:43–9. 21135881 10.1038/nrrheum.2010.197

[6] Bannuru RR Mcalindon TE Sullivan MC. Effectiveness and implications of alternative placebo treatments: a systematic review and network meta-analysis of osteoarthritis trials. Ann Intern Med. 2015;163:365–72. 26215539 10.7326/M15-0623

[7] Jones IA Togashi R Wilson ML. Intra-articular treatment options for knee osteoarthritis. Nat Rev Rheumatol. 2019;15:77–90. 30498258 10.1038/s41584-018-0123-4 PMC6390843

[8] Ahn SK Hwang JW. Global trends in immunotherapy research on breast cancer over the past 10 years. J Oncol 2020;2020:4708394. 33204263 10.1155/2020/4708394 PMC7661143

[9] Chen C. Searching for intellectual turning points: progressive knowledge domain visualization. Proc Natl Acad Sci U S A. 2004;101(Suppl 1):5303–10. 14724295 10.1073/pnas.0307513100 PMC387312

[10] Ma L Ma J Teng M. Visual analysis of colorectal cancer immunotherapy: a bibliometric analysis from 2012 to 2021. Front Immunol. 2022;13:843106. 35432385 10.3389/fimmu.2022.843106 PMC9009266

[11] Chen H Li R Zhang F. A scientometric visualization analysis for natural products on cancer research from 2008 to 2020. Front Pharmacol. 2021;12:650141. 34421584 10.3389/fphar.2021.650141 PMC8377543

[12] Zhou T Yuan Z Weng J. Challenges and advances in clinical applications of mesenchymal stromal cells. J Hematol Oncol 2021;14:24. 33579329 10.1186/s13045-021-01037-x PMC7880217

[13] Galipeau J. Macrophages at the nexus of mesenchymal stromal cell potency: the emerging role of chemokine cooperativity. Stem Cells. 2021;39:1145–54. 33786935 10.1002/stem.3380 PMC8453730

[14] Wiggers TG Winters M Van den Boom NA. Autologous stem cell therapy in knee osteoarthritis: a systematic review of randomised controlled trials. Br J Sports Med. 2021;55:1161–9. 34039582 10.1136/bjsports-2020-103671

[15] Lamo-Espinosa JM Prosper F Blanco JF. Long-term efficacy of autologous bone marrow mesenchymal stromal cells for treatment of knee osteoarthritis. J Transl Med. 2021;19:506. 34895259 10.1186/s12967-021-03160-2 PMC8666077

[16] Doyle EC Wragg NM Wilson SL. Intraarticular injection of bone marrow-derived mesenchymal stem cells enhances regeneration in knee osteoarthritis. Knee Surg Sports Traumatol Arthrosc. 2020;28:3827–42. 32006075 10.1007/s00167-020-05859-z PMC7669782

[17] Wang Y Fang J Liu B. Reciprocal regulation of mesenchymal stem cells and immune responses. Cell Stem Cell. 2022;29:1515–30. 36332569 10.1016/j.stem.2022.10.001

[18] Bennell KL Hunter DJ Paterson KL. Platelet-Rich plasma for the management of hip and knee osteoarthritis. Curr Rheumatol Rep. 2017;19:24. 28386761 10.1007/s11926-017-0652-x

[19] Szwedowski D Szczepanek J Paczesny L. The effect of Platelet-Rich plasma on the Intra-Articular microenvironment in knee osteoarthritis. Int J Mol Sci . 2021;22:5492. 34071037 10.3390/ijms22115492 PMC8197096

[20] Andia I Maffulli N. Platelet-rich plasma for managing pain and inflammation in osteoarthritis. Nat Rev Rheumatol. 2013;9:721–30. 24080861 10.1038/nrrheum.2013.141

[21] Cook CS Smith PA. Clinical update: why PRP should be your first choice for injection therapy in treating osteoarthritis of the knee. Curr Rev Musculoskelet Med. 2018;11:583–92. 30350299 10.1007/s12178-018-9524-x PMC6220006

[22] Bennell KL Paterson KL Metcalf BR. Effect of intra-articular Platelet-Rich plasma vs placebo injection on pain and medial tibial cartilage volume in patients with knee osteoarthritis: The RESTORE randomized clinical trial. JAMA. 2021;326:2021–30. 34812863 10.1001/jama.2021.19415 PMC8611484

[23] Belk JW Kraeutler MJ Houck DA. Platelet-Rich plasma versus hyaluronic acid for knee osteoarthritis: a systematic review and meta-analysis of randomized controlled trials. Am J Sports Med. 2021;49:249–60. 32302218 10.1177/0363546520909397

[24] Filardo G Kon E Pereira RM. Platelet-rich plasma intra-articular injections for cartilage degeneration and osteoarthritis: Single- versus double-spinning approach. Knee Surg Sports Traumatol Arthrosc. 2012;20:2082–91. 22203046 10.1007/s00167-011-1837-x

[25] Yin WJ Xu HT Sheng JG. Advantages of pure Platelet-Rich plasma compared with leukocyte- and Platelet-Rich plasma in treating rabbit knee osteoarthritis. Med Sci Monit. 2016;22:1280–90. 27086145 10.12659/MSM.898218 PMC4837928

[26] Di Martino A Boffa A Andriolo L. Leukocyte-Rich versus Leukocyte-Poor Platelet-Rich plasma for the treatment of knee osteoarthritis: a Double-Blind randomized trial. Am J Sports Med. 2022;50:609–17. 35103547 10.1177/03635465211064303

[27] Chahla J Cinque ME Piuzzi NS. A call for standardization in Platelet-Rich plasma preparation protocols and composition reporting: a systematic review of the clinical orthopaedic literature. J Bone Joint Surg Am. 2017;99:1769–79. 29040132 10.2106/JBJS.16.01374

[28] Pereira TV Juni P Saadat P. Viscosupplementation for knee osteoarthritis: systematic review and meta-analysis. BMJ 2022;378:e069722. 36333100 10.1136/bmj-2022-069722 PMC9258606

[29] Jevsevar DS Brown GA Jones DL. The American Academy of Orthopaedic Surgeons evidence-based guideline on: treatment of osteoarthritis of the knee, 2nd edition. J Bone Joint Surg Am. 2013;95:1885–6. 24288804 10.2106/00004623-201310160-00010

[30] Wang CC Wang CT Chou WC. Hyaluronic acid injection reduces inflammatory and apoptotic markers through modulation of AKT by repressing the oxidative status of neutrophils from osteoarthritic synovial fluid. Int J Biol Macromol. 2020;165:2765–72. 33736281 10.1016/j.ijbiomac.2020.10.154

[31] Ghosh P Guidolin D. Potential mechanism of action of intra-articular hyaluronan therapy in osteoarthritis: are the effects molecular weight dependent? Semin Arthritis Rheum. 2002;32:10–37. 12219318 10.1053/sarh.2002.33720

[32] Carlson VR Ong AC Orozco FR. Compliance with the AAOS guidelines for treatment of osteoarthritis of the knee: a survey of the american association of hip and knee surgeons. J Am Acad Orthop Surg. 2018;26:103–7. 29283898 10.5435/JAAOS-D-17-00164

[33] Bannuru RR Natov NS Dasi UR. Therapeutic trajectory following intra-articular hyaluronic acid injection in knee osteoarthritis--meta-analysis. Osteoarthritis Cartilage. 2011;19:611–9. 21443958 10.1016/j.joca.2010.09.014 PMC11678314

[34] Moser LB Bauer C Jeyakumar V. Hyaluronic acid as a carrier supports the effects of glucocorticoids and diminishes the cytotoxic effects of local anesthetics in human articular chondrocytes in vitro. Int J Mol Sci . 2021;22:11503. 34768933 10.3390/ijms222111503 PMC8583767

[35] Nishida Y Kano K Nobuoka Y. Efficacy and safety of Diclofenac-Hyaluronate conjugate (Diclofenac etalhyaluronate) for knee osteoarthritis: a randomized phase III trial in japan. Arthritis Rheumatol. 2021;73:1646–55. 33749997 10.1002/art.41725 PMC8456865

[36] Kijowski R Demehri S Roemer F. Osteoarthritis year in review 2019: imaging. Osteoarthritis Cartilage. 2020;28:285–95. 31877380 10.1016/j.joca.2019.11.009

[37] Hu W Chen Y Dou C. Microenvironment in subchondral bone: predominant regulator for the treatment of osteoarthritis. Ann Rheum Dis. 2021;80:413–22. 33158879 10.1136/annrheumdis-2020-218089 PMC7958096

[38] Suri S Walsh DA. Osteochondral alterations in osteoarthritis. Bone. 2012;51:204–11. 22023932 10.1016/j.bone.2011.10.010

[39] Cabahug-Zuckerman P Frikha-Benayed D Majeska RJ. Osteocyte apoptosis caused by hindlimb unloading is required to trigger osteocyte RANKL production and subsequent resorption of cortical and trabecular bone in mice femurs. J Bone Miner Res. 2016;31:1356–65. 26852281 10.1002/jbmr.2807 PMC5488280

[40] Zhou X Cao H Yuan Y. Biochemical signals mediate the crosstalk between cartilage and bone in osteoarthritis. Biomed Res Int. 2020;2020:5720360. 32337258 10.1155/2020/5720360 PMC7165323

[41] Mcalindon TE Bannuru RR Sullivan MC. OARSI guidelines for the non-surgical management of knee osteoarthritis. Osteoarthritis Cartilage. 2014;22:363–88. 24462672 10.1016/j.joca.2014.01.003

[42] Sellam J Berenbaum F. The role of synovitis in pathophysiology and clinical symptoms of osteoarthritis. Nat Rev Rheumatol. 2010;6:625–35. 20924410 10.1038/nrrheum.2010.159

[43] Koenig KM Ong KL Lau EC. The use of hyaluronic acid and corticosteroid injections among medicare patients with knee osteoarthritis. J Arthroplasty. 2016;31:351–5. 26421601 10.1016/j.arth.2015.08.024

[44] Hochberg MC Altman RD April KT. American College of Rheumatology 2012 recommendations for the use of nonpharmacologic and pharmacologic therapies in osteoarthritis of the hand, hip, and knee. Arthritis Care Res (Hoboken). 2012;64:465–74. 22563589 10.1002/acr.21596

[45] Katz JN Arant KR Loeser RF. Diagnosis and treatment of hip and knee osteoarthritis: a review. JAMA. 2021;325:568–78. 33560326 10.1001/jama.2020.22171 PMC8225295

[46] Conaghan PG Cohen SB Berenbaum F. Brief report: a phase IIb trial of a novel Extended-Release microsphere formulation of triamcinolone acetonide for intraarticular injection in knee osteoarthritis. Arthritis Rheumatol 2018;70:204–11. 29088579 10.1002/art.40364 PMC5814922

[47] Gowler P Turnbull J Shahtaheri M. Interplay between cellular changes in the knee joint, circulating lipids and pain behaviours in a slowly progressing murine model of osteoarthritis. Eur J Pain. 2022;26:2213–26. 36097797 10.1002/ejp.2036 PMC9826505

[48] Mcalindon TE Lavalley MP Harvey WF. Effect of intra-articular triamcinolone vs saline on knee cartilage volume and pain in patients with knee osteoarthritis: a randomized clinical trial. JAMA. 2017;317:1967–75. 28510679 10.1001/jama.2017.5283 PMC5815012

[49] Amos N Lauder S Evans A. Adenoviral gene transfer into osteoarthritis synovial cells using the endogenous inhibitor IkappaBalpha reveals that most, but not all, inflammatory and destructive mediators are NFkappaB dependent. Rheumatology (Oxford). 2006;45:1201–9. 16571608 10.1093/rheumatology/kel078

[50] Jia Y He W Zhang H. Morusin ameliorates IL-1beta-Induced chondrocyte inflammation and osteoarthritis via NF-kappaB signal pathway. Drug Des Devel Ther. 2020;14:1227–40. 10.2147/DDDT.S244462 PMC7105369 32273685

[51] Arra M Swarnkar G Ke K. LDHA-mediated ROS generation in chondrocytes is a potential therapeutic target for osteoarthritis. Nat Commun. 2020;11:3427. 32647171 10.1038/s41467-020-17242-0 PMC7347613

[52] Di Martino A Di Matteo B Papio T. Platelet-Rich plasma versus hyaluronic acid injections for the treatment of knee osteoarthritis: results at 5 years of a Double-Blind, randomized controlled trial. Am J Sports Med. 2019;47:347–54. 30545242 10.1177/0363546518814532

[53] Lin KY Yang CC Hsu CJ. Intra-articular injection of Platelet-Rich plasma is superior to hyaluronic acid or saline solution in the treatment of mild to moderate knee osteoarthritis: a randomized, Double-Blind, Triple-Parallel, Placebo-Controlled clinical trial. Arthroscopy. 2019;35:106–17. 30611335 10.1016/j.arthro.2018.06.035

[54] Choi MH Blanco A Stealey S. Micro-Clotting of Platelet-Rich plasma upon loading in hydrogel microspheres leads to prolonged protein release and slower microsphere degradation. Polymers (Basel). 2020;12:1712. 32751604 10.3390/polym12081712 PMC7464943

[55] Ma M Zou F Abudureheman B. Magnetic microcarriers with accurate localization and proliferation of mesenchymal stem cell for cartilage defects repairing. ACS Nano. 2023;17:6373–86. 36961738 10.1021/acsnano.2c10995

[56] Lee WS Kim HJ Kim KI. Intra-Articular injection of autologous adipose Tissue-Derived mesenchymal stem cells for the treatment of knee osteoarthritis: a phase IIb, randomized, Placebo-Controlled clinical trial. Stem Cells Transl Med. 2019;8:504–11. 30835956 10.1002/sctm.18-0122 PMC6525553

[57] Freitag J Bates D Wickham J. Adipose-derived mesenchymal stem cell therapy in the treatment of knee osteoarthritis: a randomized controlled trial. Regen Med. 2019;14:213–30. 30762487 10.2217/rme-2018-0161

